# Prevalence of Thyroid Dysfunction in Newly Diagnosed Rheumatoid Arthritis Patients

**DOI:** 10.7759/cureus.18204

**Published:** 2021-09-23

**Authors:** Kefayatullah Nazary, Nabeel Hussain, Roland Oluwapelumi Ojo, Sana Anwar, Firas Kadurei, Farukhzad Hafizyar, Daniya Muhammad Haroon, Rakesh Khemani, Abdul Subhan Talpur

**Affiliations:** 1 Internal Medicine, Kabul University of Medical Sciences, Kabul, AFG; 2 Internal Medicine, Saba University School of Medicine, Devens, USA; 3 Internal Medicine, College of Medicine, University of Lagos, Lagos, NGA; 4 Internal Medicine, Lugansk State Medical University, Luhansk, UKR; 5 Internal Medicine, Mubarak Al-Kabeer Hospital, Jabriya, KWT; 6 Internal Medicine, Ariana Sabet Hospital, Kabul, AFG; 7 Internal Medicine, Bahria University Medical & Dental College, Karachi, PAK; 8 Internal Medicine, Jinnah Sindh Medical University, Karachi, PAK; 9 Internal Medicine, Liaquat University of Medical and Health Sciences, Jamshoro, PAK

**Keywords:** rheumatoid arthritis, auto immune, hypothyroidism, hyperthyroidism, subclinical hypothyroidism

## Abstract

Introduction: Rheumatoid arthritis (RA) is associated with various autoimmune disorders, including thyroid dysfunction. However, local data studying the prevalence of thyroid dysfunction in newly diagnosed RA patients are limited.

Methods: This case-control study was conducted between January 2019 to December 2020 in the Internal Medicine Department of Liaquat University of Medical and Health Sciences. The study group of 400 newly diagnosed patients with RA was enrolled in the study. Another 400 patients without the diagnosis of RA, adjusted for age and gender, were enrolled in the study as a control group and their thyroid functions were compared.

Results: Patients with RA had more participants with thyroid dysfunction compared to patients without RA (25.25% vs. 11.5%; p-value: 0.00001). In addition, more patients with RA had concomitant primary hypothyroidism compared to the control group (7.75% vs. 2.5%; p-value: 0.0007). Furthermore, patients with RA also had a higher prevalence of subclinical hypothyroidism (13.0% vs. 5.5%; p-value: 0.0002).

Conclusion: Our study indicates that thyroid dysfunction is significantly prevalent in patients with RA. Based on our findings, it is suggested that management and follow-up of RA patients should include the screening of thyroid auto-antibodies and thyroid dysfunction.

## Introduction

Rheumatoid arthritis (RA) is an autoimmune disorder that results in chronic inflammation of the joints leading to consecutive joint destruction, which results in reduced mobility and increased disability [1]. In the general population, its prevalence is ∼1%, and it is found to be linked with some comorbidities [2]. The dysfunction of the thyroid includes hyperthyroidism and hypothyroidism.

Hyperthyroidism is defined as the excessive secretion of thyroid hormones, and its most common etiology is Graves’ disease. Hypothyroidism is defined as a condition where the thyroid gland does not release sufficient thyroid hormones into the bloodstream, and its most common etiology is Hashimoto’s thyroiditis. Both the types are further categorized into overt and subclinical stages [3]. Both hyperthyroidism and hypothyroidism have adverse effects on human health and can result in an increased risk of cardiovascular diseases and mortality. Previous studies demonstrated the prevalence of thyroid dysfunction ranging from 6% to 34% in RA patients [4,5]. Thyroid assessment is recommended in various autoimmune disorders [6]. The thyroid assessment includes thyroid function tests (TFTs) along with clinical symptoms like cold intolerance, loss of weight, elevated metabolism, or thyroid goiter [3]. However, TFTs are not recommended in RA patients.

There are very limited data available on the association between RA patients and thyroid function, particularly in the South Asian region. Therefore, this study aims to further investigate the prevalence of thyroid dysfunction in RA patients.

## Materials and methods

This case-control study was conducted between January 2019 to December 2020 in the Internal Medicine Department of Liaquat University of Medical and Health Sciences. The study group of 400 newly diagnosed patients with RA was enrolled in the study. These patients presented to the outpatient department (OPD) with complaints of fever, fatigue, joint pain, tenderness, swelling, and stiffness. The diagnosis of RA was made on the basis of clinical symptoms such as joint pain, X-ray findings such as bony erosions, soft tissue swelling, and joint space narrowing, and the presence of anti-cyclic citrullinated peptide (anti-CCP). Ethical review board approval was taken from Liaquat University of Medical and Health Sciences (LUMHS/IRB-OFC/2019-12-02). Another 400 participants, mainly the attendants accompanying patients, without the diagnosis of RA and adjusted for age and gender, were enrolled in the study as a control group. Exclusion criteria included patients with a prior diagnosis of thyroid disorder, patients who had recent surgery or major illness such as myocardial infarction or stroke, and patients who were on medications known to influence thyroid hormones such as metformin, interferon, amiodarone, and anti-epileptics.

After the process of enrollment was completed, each patient's venous blood sample was taken from the cubital vein and sent to the laboratory for thyroid hormone assessment. Serum free thyroxine (FT4) was determined by radioimmunoassay (RIA) and thyroid-stimulating hormone (TSH) was evaluated by immunoradiometric assay (IRMA) techniques using commercial kits of Immunotech Inc. (Beckman, Czech Republic). Normal ranges for FT4, free triiodothyronine (FT3), and TSH, as standardized in our laboratory, are 11.0-22.0 pmol/L, 2.5-5.8 pmol/L, and 0.3-4.0 mIU/L, respectively. Thyroid dysfunctions were labeled based on Table 1.

**Table 1:** FT4 and TSH levels in thyroid dysfunction. FT4, free thyroxine; TSH, thyroid-stimulating hormone.

Thyroid dysfunction	FT4	TSH
Primary hyperthyroidism	Increased	Decreased
Primary hypothyroidism	Decreased	Increased
Subclinical hypothyroidism	Normal	Increased
Subclinical hyperthyroidism	Normal	Decreased

Data were analyzed using the Statistical Package for the Social Sciences (SPSS v. 21.0) (IBM Corp, Armonk, New York). Numerical data were represented by mean and standard deviation, while categorical data were tabulated as frequency and percentages. Unpaired t-test was used to compare numerical values, while chi-square test was used to compare categorical values between patients with and without RA. A p-value lower than 0.05 meant that the difference between the case group and control group was significant and the null hypothesis was not valid.

## Results

The mean age of RA patients in the case group was found to be 36 ± 08 years, whereas that of the control group was 37 ± 07 years; however, the difference was not significant. Thyroid hormone values were also compared between the two groups and no significant difference was found (Table 2).

**Table 2:** Comparison of characteristics and TFTs of both groups. FT4, free thyroxine; mIU/L, milli-international units per liter; NS, nonsignificant; pmol/L, picomole per liter; TFT, thyroid function test; TSH, thyroid-stimulating hormone.

Characteristics	Case group (n = 400)	Control group (n = 400)	p-value
Age in years	36 ± 08	37 ± 07	NS
Gender			
Male	159	164	NS
Female	241	236	NS
Mean values			
FT4 (pmol/L)	17.2 ± 4.7	17.9 ± 4.5	NS
TSH (mIU/L)	2.0 ± 0.7	1.9 ± 0.6	NS

Patients with RA had more participants with thyroid dysfunction compared to patients without RA (25.25% vs. 11.5%; p-value: 0.00001). In addition, more patients with RA had concomitant primary hypothyroidism compared to the control group (7.75% vs. 2.5%; p-value: 0.0007). Furthermore, patients with RA also had a higher prevalence of subclinical hypothyroidism (13.0% vs. 5.5%; p-value: 0.0002) (Table 3).

**Table 3:** Thyroid dysfunction in case and control groups. NS, nonsignificant.

Thyroid dysfunction	Case group (n = 400)	Control group (n = 400)	p-value
Euthyroid	299 (74.75%)	354 (88.5%)	0.00001
Primary hyperthyroidism	08 (2.0%)	06 (1.5%)	NS
Primary hypothyroidism	31 (7.75%)	10 (2.5%)	0.0007
Subclinical hyperthyroidism	10 (2.5%)	08 (2.0%)	NS
Subclinical hypothyroidism	52 (13.0%)	22 (5.5%)	0.0002

## Discussion

Thyroid dysfunction, particularly primary hypothyroidism and subclinical hypothyroidism, was prevalent in patients with RA compared to patients without RA in our study. Furthermore, 25.5% of RA patients had thyroid dysfunction compared to 11.5% in the control group. Our result was comparable to the findings of other studies. Li et al. indicated a significantly higher prevalence of thyroid dysfunction in RA patients than in controls (32.3% vs. 14.2%; p-value: <0.001) [1]. However, both hyperthyroidism and hypothyroidism were significantly more prevalent in the RA group, compared to our study, which only reported a significant prevalence of hypothyroidism [1]. Elattar et al. reported primary hypothyroidism as the most common thyroid dysfunction in RA patients, followed by subclinical hypothyroidism, which coincides with our results [7]. Other studies have also reported an association between RA and thyroid antibodies. Andonopoulos et al. reported a difference in the level of thyroid autoantibodies in RA patients when compared to the control group [8]. Acay et al. indicated that thyroid autoantibodies are significantly more common in RA compared to the general population [9].

Since both thyroid disorder and RA are autoimmune in nature, their origin may be similar. However, the exact mechanism is not yet understood. It is believed that genetic and environmental factors are involved in the association between RA and thyroid dysfunction [10-12]. It is important to assess thyroid functions in RA, as treatment of RA may further exacerbate thyroid dysfunction. Glucocorticoid, which is used to suppress inflammation in RA patients, in high doses can lead to direct suppression of TSH secretion without increasing FT3 and FT4 [13]. Another drug, leflunomide, could also affect thyroid function [14]. Hence, various studies have suggested that thyroid screening should be routinely performed in patients with RA [15].

To the best of our knowledge, this is the first study in a local setting to study the association between RA and thyroid function. However, the study has the following limitations. First, since the study was conducted in a single institute, the sample size was very limited. Secondly, thyroid hormones were only evaluated at the time of enrollment, and not during the course of the disease. Hence, we do not know the impact of rheumatoid arthritis on thyroid over the course of time. Third, the impact of thyroid dysfunction on the treatment of RA was not evaluated.

## Conclusions

Our study indicates that thyroid dysfunction is quite prevalent in patients with RA. Based on our findings, it is suggested that management and follow-up of RA patients should include the screening of thyroid auto-antibodies and thyroid dysfunction. Further studies to understand the mechanism of association between thyroid function and RA should be conducted.
